# Toward cell transfer immunotherapy against patient-specific mutations in gastrointestinal cancers

**DOI:** 10.1186/2051-1426-2-S3-O3

**Published:** 2014-11-06

**Authors:** Eric Tran, Mojgan Ahmadzadeh, Alena Gros, Simon Turcotte, Paul F Robbins, Yong Chen Lu, Yong Li, John Wunderlich, Jared Gartner, Katherine Hogan, James Yang, Steven Rosenberg

**Affiliations:** 1 NIH/NCI/Surgery Branch, Bethesda, MD, United States; 2 Université de Montréal, and Institut du Cancer de Montréal, Centre de Recherche du Centre Hospitalier de l'Université de Montréal, Quebec, United States; 3 National Institutes of Health/ National Cancer Institute, Bethesda, MD, United States

## 

Accumulating evidence suggests that the efficacy of ACT in melanoma is mediated by T cells that target somatic mutations expressed by the patients' tumors. Moreover, in one patient with metastatic epithelial (bile duct) cancer, we recently identified tumor-infiltrating Th1 cells that specifically recognized a mutation in ERBB2IP expressed by the patient's tumors and observed tumor regression after adoptive transfer of a highly enriched population of ERBB2IP mutation-specific Th1 cells. Thus, we hypothesize that mutation-specific T cells may be frequently elicited in patients with metastatic gastrointestinal (GI) cancers and may be harnessed for ACT. To test this, we first performed whole-exome sequencing on metastatic lesions from GI cancer patients to identify the mutations. Next, we generated mingene constructs that encoded each mutation and transfected these minigenes into autologous antigen presenting cells (APCs) to allow for the processing and presentation of all the mutations expressed by the tumor. These APCs were then co-cultured with tumor infiltrating lymphocytes (TIL) and T cell reactivity against the mutations was determined by IFN-γ ELISPOT and 4-1BB and OX40 upregulation by flow cytometry. In one patient with colon cancer, 119 mutations were evaluated for mutation reactivity. Several, but not all, TIL cultures were found to contain highly variable proportions of CD8+ T cells that specifically recognized a mutation in CASP8 (67 F→V). Intriguingly, upon further expansion in vitro, these mutation-reactive CD8+ T cells were markedly outgrown by other cells in culture. In another patient with rectal cancer, 155 mutations were evaluated for mutation reactivity. We found at least 3 different mutation reactivities, two comprising CD8+ T cell responses and one CD4+ T cell response. In a third patient (cholangiocarcinoma), we did not detect T cells reactive against 38 mutations tested. For this patient, the "mutation call" threshold has been lowered and an additional 125 putative mutations will be evaluated. Thus, our preliminary data suggests that the ability of the human immune system to mount a T cell response against somatic mutations in metastatic GI cancers may not be a rare event. Current efforts are focused on evaluating more patients for mutation-specific T cell responses and on developing methods to enrich mutation-specific T cells or T cell receptors for therapy, which may provide a path to extend cell transfer immunotherapy to almost all common solid cancers.

## Consent

Written informed consent was obtained from the patient for publication of this abstract and any accompanying images. A copy of the written consent is available for review by the Editor of this journal.

